# Correction: Hydrogen production by electrochemical reaction using ethylene glycol with terephthalic acid

**DOI:** 10.1039/d1ra90029c

**Published:** 2021-01-28

**Authors:** Se-Hyun Kim, Sang-Won Woo, Chan-Soo Kim, Sung-Eun Lee, Tae-Oh Kim

**Affiliations:** Department of Environmental Engineering, Kumoh National Institute of Technology Gumi 39177 Republic of Korea tokim@kumoh.ac.kr; Marine Energy Convergence & Integration Laboratory, Jeju Global Research Center, Korea Institute of Energy Research Jeju Republic of Korea; Department of Applied Biosciences, Kyungpook National University Daegu 41566 Republic of Korea selpest@knu.ac.kr; Department of Energy Engineering Convergence, Kumoh National Institute of Technology Gumi 39177 Republic of Korea

## Abstract

Correction for ‘Hydrogen production by electrochemical reaction using ethylene glycol with terephthalic acid’ by Se-Hyun Kim *et al.*, *RSC Adv.*, 2021, **11**, 2088–2095, DOI: 10.1039/D0RA10187G.

The authors regret the omission of a funding acknowledgement in the original article. This acknowledgement is given below.

This work was supported by a National Research Foundation of Korea (NRF) grant, funded by the Korean Government (NRF2016R1D1A1A09918845).

The Royal Society of Chemistry apologises for these errors and any consequent inconvenience to authors and readers.

## Supplementary Material

